# Delayed Acetaminophen Absorption Resulting in Acute Liver Failure

**DOI:** 10.1155/2022/3672248

**Published:** 2022-05-07

**Authors:** Huiling Tan, Paul Stathakis, Benoj Varghese, Nicholas A. Buckley, Angela L. Chiew

**Affiliations:** ^1^Department of Critical Care Medicine, Royal Hobart Hospital, Hobart, Tasmania, Australia; ^2^NSW Health Pathology, Prince of Wales Hospital, Randwick, NSW, Australia; ^3^New South Wales Poisons Information Centre, Children's Hospital at Westmead, NSW, Australia

## Abstract

*Introduction*. Acetaminophen is a common medication involved in deliberate and accidental self-poisoning. The acetaminophen treatment nomogram is used to guide acetylcysteine treatment. It is rare to develop hepatotoxicity with an initial acetaminophen concentration below the nomogram line. We present a case of acetaminophen ingestion with an initial concentration below the nomogram line that developed hepatic failure, due to a delayed peak acetaminophen concentration secondary to coingesting medications that slow gastric emptying. *Case Report*. A 43-year-old (55 kg) female presented after ingesting an unknown quantity of acetaminophen, clonidine, and alcohol. Her acetaminophen level was 41 mg/L (256 *μ*mol/L) at 4.5 h post-ingestion, well below the nomogram line, and ALT was 25 U/L. Hence, acetylcysteine was not commenced. She was intubated for decreased level of conscious. A repeat acetaminophen level 4 h later was 39 mg/L (242 *μ*mol/L), still below the nomogram line. She was extubated 24 h later.At 38 h post-ingestion she developed abdominal pain, the repeat acetaminophen level was 85 mg/L (560 *μ*mol/L), ALT was 489 U/L, and acetylcysteine was commenced. The patient developed hepatic failure with a peak ALT of 7009 U/L and INR of 7.5 but made a full recovery. It was discovered that she had ingested a combination acetaminophen product containing dextromethorphan and chlorphenamine. Acetaminophen metabolites were measured, including nontoxic glucuronide and sulfate conjugates and toxic cytochrome P450 (CYP) metabolites. The metabolite data demonstrated increasing CYP metabolites in occurrence with the delayed acetaminophen peak concentration. *Discussion*. Opioids and antimuscarinic agents are known to delay gastric emptying and clonidine may also have contributed. These coingested medications resulted in delayed acetaminophen absorption. This case highlights the issue of altered pharmacokinetics when patients coingest gut slowing agents.

## 1. Introduction

Acetaminophen is a common medication taken in deliberate self-poisoning due to its wide availability. After an acute ingestion, it is generally absorbed within hours, and the peak serum concentration typically occurs within 4 hours of ingestion. The serum concentration then decreases based on the elimination half-life. Acute ingestions are decontaminated with activated charcoal if the hospital presentation is within 2 hours of ingestion [1], and intravenous acetylcysteine is given to those with an acetaminophen concentration above the treatment nomogram line [2]. The risk of liver injury is very low in those below the nomogram line or who receive acetylcysteine within 8 hours of exposure [3].

We present a case of acute acetaminophen ingestion with an initial acetaminophen concentration well below the treatment nomogram line and very delayed absorption and peak acetaminophen concentrations, which resulted in fulminant hepatic failure.

## 2. Case

A 43-year-old (55 kg) female, presented to the emergency department following a polypharmacy overdose, believed to be of an unknown quantity of acetaminophen, clonidine, and alcohol. She had been last seen well 4 hours prior to her hospital presentation, and this was presumed to be her time of ingestion. She had a background of previous self-harm, anorexia nervosa, and alcohol misuse (half a bottle of vodka every night). Her regular medications included clonidine, thiamine, and a multivitamin.

**Figure 1:**
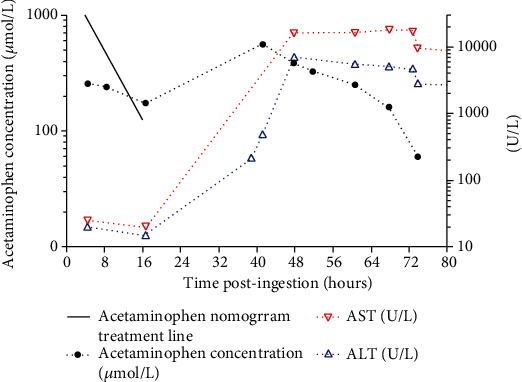
Acetaminophen concentrations, ALT, and AST vs. time post-ingestion. Note: 16 h acetaminophen concentration was measured retrospectively after the patient developed hepatotoxicity.

On admission, her respiratory rate was 8 bpm, oxygen saturation 99% on room air, blood pressure of 98/62 mmHg, heart rate of 50 bpm, and Glasgow coma score of 3. She showed signs of significant clonidine toxicity (reduced conscious state and bradycardia [lowest heart rate = 45 bpm]). Hence, she was intubated for airway protection, but no gastrointestinal decontamination (i.e., activated charcoal or gastric lavage) was performed. Her initial bloods revealed AST 25 IU/L (RR: 5-30 IU/L), ALT 20 IU/L (RR: 5-35 IU/L), INR 1.1 (RR: 0.9-1.2), lactate 2.1 mmol/L (RR: < 2 mmol/L), and ethanol level 0.37%. Her acetaminophen level was 41 mg/L (256 *μ*mol/L). The family indicated the earliest and latest possible time of ingestion was 4.5 hours and 45 minutes prior to presentation, respectively. The acetaminophen level was well below the treatment nomogram line, timed from the earliest possible time of ingestion of 4.5 h (Figure 1). Acetylcysteine was not commenced, and her acetaminophen level was repeated 4 h later and was 39 mg/L (242 *μ*mol/L). This remained well below the nomogram line at 8.5 h (presumed earliest possible time of ingestion) (Figure 1). She remained hemodynamically stable and was extubated the following morning, where she remained in the intensive care unit (ICU) for observation. At 38 h post-ingestion, she developed generalized abdominal pain, hypotension (mean arterial pressure [MAP] 52 mmHg), and a rising lactate (5.9 mmol/L) despite fluid resuscitation. At this stage, she had a CT abdomen and pelvis, which demonstrated generalized mesenteric edema, pericholecystic fluid, periportal edema, and edematous walls of the entire small and large bowel with reduced enhancement in keeping with pancolitis. Repeated pathology at 38 hours post-ingestion revealed an acute liver injury (ALI) with an ALT of 489 U/L, INR of 1.5, and acetaminophen level of 85 mg/L (560 *μ*mol/L) (Figure 1). The impression of the general surgical team was her clinical picture is not consistent with an acute surgical pathology, and no further imaging was performed during her admission. Acetylcysteine (300 mg/kg IV over 20 h) was commenced at 46 h post-ingestion, and she developed a noradrenaline requirement at 47 h post-ingestion for a MAP of 55 mmHg. She subsequently developed fulminant hepatic failure with oliguric acute kidney injury requiring continuous renal replacement therapy with a peak ALT of 7009 U/L, peak INR of 7.5, and peak creatinine of 138 *μ*mol/L (Supplementary Table 1: patient's pathology results during admission). Her case was discussed with the liver transplant team, but she was not deemed suitable for liver transplant. During her stay in ICU, she developed mild encephalopathy, which did not require reintubation. Her other interventions include continuous acetylcysteine infusion and continuous renal replacement therapy for 19 days. Despite a prolonged stay in the ICU for a month, her liver and kidney function returned to baseline, and she was discharged back to the community.

On further interview with the patient, she stated that she was unable to recall the exact events leading up to her presentation. While in ICU, she was closely monitored by the medical staff with no access to acetaminophen. Her husband subsequently revealed that the acetaminophen tablets the patient ingested were red and white capsules, which were bought over the counter from a pharmacy. A search of available products in Australia found that the only red and white capsules containing acetaminophen are a combination over the counter cold and flu tablet containing acetaminophen, dextromethorphan, and chlorphenamine [4].

**Figure 2:**
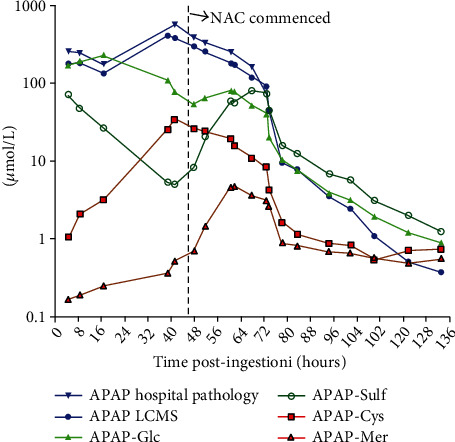
Acetaminophen and metabolite concentrations vs. time post-ingestion.

Consent was obtained from the patient to measure acetaminophen metabolite concentrations and case report publication. These included nontoxic glucuronide (APAP-Glu) and sulfate (APAP-Sul) conjugates and adducts of the toxic metabolite N-acetyl-p-benzoquinone imine (NAPQI), including acetaminophen-cysteine (APAP-Cys) and acetaminophen-mercapturate (APAP-Mer) (Figure 2). Acetaminophen metabolite concentrations were measured with an AB SCIEX Triple Quad™ 5500 liquid chromatography with tandem mass spectrometry system (derived from An et al. (2011)) using both positive ionization mode for APAP and APAP-d4 (internal standard) and negative ionization mode for APAP-Glc, APAP-Sul, APAP-Cys, APAP-Mer, and APAP-Glc-d3 (internal standard) [5]. The LC-MS/MS analysis was performed at South-Eastern Area Laboratory Services, Prince of Wales Hospital, Sydney.

The area under the curve (AUC) was calculated for each acetaminophen metabolite. Acetaminophen and metabolite concentrations are shown in molar units, so they are comparable. All analyses were performed using GraphPad PRISM® software version 8.0.2. The AUC for each metabolite was compared to the sum of AUC of all metabolites. APAP-Glu and APAP-Sul accounted for 68% (AUC 8663 (*μ*mol/L)∗hr) and 22% (AUC 2733 (*μ*mol/L)∗hr) of the total metabolites, respectively, while the metabolites APAP-Cys and APAP-Mer reflect the metabolism of acetaminophen by the cytochrome P450 (CYP) enzyme system, combined accounted for 10% (AUC 1254 (*μ*mol/L)∗hr) of the total metabolites (Figure 2).

## 3. Discussion

We present a case of hepatotoxicity and subsequent acute liver failure in a patient with very delayed absorption and peak acetaminophen concentration with two earlier acetaminophen concentrations well below the acetaminophen treatment nomogram line. We also present the acetaminophen metabolite data, which demonstrates the increasing CYP metabolites coinciding with the delayed higher acetaminophen concentrations.

Rare cases of hepatotoxicity have been reported despite an initial acetaminophen concentration below the nomogram line [6–8]. Higher risk patients include those with a history of starvation or malnutrition, chronic use of enzyme inducing drugs, a history of chronic alcohol use, and chronic debilitating disease. This patient had a history of chronic alcohol use and an eating disorder. In the Canadian Acetaminophen Overdose Study (CAOS), they found 131/1215 (10.8%) with an initial acetaminophen concentration below the standard treatment line (150 mg/L at 4 hours) crossed over this line at some later time; 65 (5.3%) of these also crossed the 200 mg/L at 4 hours line. Two of these line-crossers developed hepatotoxicity. The factors associated with line-crossing were older age, male sex, and coingestion of NSAIDS, opioids, or antimuscarinics [9].

Our case was unusual as absorption was extremely delayed, and she did not cross the treatment nomogram line until 16 h post-ingestion. However, this acetaminophen concentration was only tested in retrospect.Hence, acetylcysteine was not commenced until the patient developed symptoms from hepatotoxicity. It is likely the coingestion of gut slowing medication resulted in delayed acetaminophen absorption. The true peak acetaminophen concentration was somewhere between 16 and 39 h post-ingestion. She did not receive activated charcoal, which might have prevented this from occurring. Acetaminophen is rapidly absorbed in the small intestine; however, absorption is dependent on dissolution and the gastric emptying rate controlling the extent to which drug is delivered to the small intestine [10]. The history was not available at the time of presentation, but she likely ingested a combination product containing dextromethorphan and chlorphenamine. These have opioid and antimuscarinic properties, respectively, which are known to delay gastric emptying. In a cohort study of patients who ingested combination acetaminophen products of opioids or antihistamines, 5/76 (6.6%) with an initial acetaminophen concentration below the 150 mg/L at 4 h nomogram line crossed the treatment line on a repeat level. Four were treated with acetylcysteine and none developed hepatotoxicity. Three other patients had repeat concentrations that either rose without crossing the line or became very close to intersecting it. None of these patients developed hepatotoxicity. The authors recommend repeating an acetaminophen concentration between 7- and 8-hour post-ingestion [11].

There have been several other reports of delayed acetaminophen peak concentration or double acetaminophen peak concentrations secondary to coingestions with agents that slow gut motility or following massive acetaminophen ingestions [8, 12–16]. This patient reported a clonidine ingestion and on presentation had evidence of clonidine (alpha2 adrenoceptor agonist) toxicity including coma and bradycardia [17]. Clonidine may decrease gastric emptying with varying results in animal and human (therapeutic doses) studies. Rat studies have demonstrated that clonidine can strongly inhibit gastrointestinal transit in the small intestine but only weakly inhibit gastric emptying [18] and may inhibit gastric emptying of indigestible solids [19]. Conversely, other animal studies have demonstrated little effect of alpha2 adrenoceptor agonists on gastric emptying [20, 21]. In contrast, oral clonidine did not significantly delay gastric emptying of either a solid meal or liquids in humans [22, 23]. However, in diabetic gastropathy patients, a therapeutic dose of clonidine showed a trend toward delayed emptying [24]. Thus, it is likely that the overdose of clonidine might also have contributed to the delayed gastric emptying and acetaminophen absorption seen in this case.

Furthermore, gastric emptying may be delayed in mechanically ventilated patients [25]. A study in ventilated intensive care patients, within the first 72 h of their ICU admission, showed delayed gastric emptying with an acetaminophen absorption model. Moreover, they found that the use of opioids is associated with impaired emptying in a dose dependent phenomenon [26]. In this case, the patient received a low dose of fentanyl and propofol whilst intubated. Changes to gastrointestinal tract motility may predispose to drug clumping or pharmacobezoar formation. Although this patient presented >4 hours post-ingestion, once intubated, she would likely still have benefited from activated charcoal.

In this case, we measured the acetaminophen metabolite concentrations. Acetaminophen in therapeutic doses in adults is mainly metabolized into nontoxic glucuronide (52–57%) and sulfate conjugates (30-44%). A small fraction (typically ~5%) is metabolized by CYP enzymes into the reactive metabolite NAPQI. NAPQI is detoxified by irreversible glutathione-dependent conjugation reactions to mercapturic acid and cysteine conjugates [27]. In overdose, the increased formation of NAPQI depletes hepatic glutathione and NAPQI covalently binds to critical cellular proteins [28]. NAPQI is primarily responsible for acetaminophen-induced hepatotoxicity. The fraction of CYP metabolites has been reported to be higher in those who develop an acute liver injury compared to those that do not [29]. This patient had a higher fraction of CYP metabolites (10%) when compared to therapeutic ingestion (<5%) or acetaminophen overdoses that do not develop liver injury [29].

Of note is the profile of the acetaminophen metabolites over time.Initially, the CYP metabolites represent approximately 1% of the total metabolites and APAP-Sulf approximately 30%. Compared to glucuronidation, which is a nonsaturable pathway except in massive ingestions [30], sulfation is considered to be a high-affinity, low-capacity saturable process [31]. Sulfation becomes saturated at therapeutic doses [32], as inorganic sulfate that provides the source of sulfate for sulfotransferase (SULT) becomes depleted [31, 33]. In this case, the APAP-Sulf concentration decreased, while the CYP metabolite increased; at 40 h post-ingestion, the CYP metabolites account for approximately 30% of total metabolites and APAP-Sulf <5%. After initiation of acetylcysteine, an increase in APAP-Sulf is seen. Acetylcysteine is a cysteine precursor that is hydrolysed intracellularly to cysteine, which replenishes glutathione [34]. Its efficacy as a specific antidote for acetaminophen poisoning relies mainly on this ability to stimulate glutathione synthesis [35]. Acetylcysteine also supplies thiol groups, which can directly bind with NAPQI in hepatocytes [36] and enhance sulfate conjugation [37]. The metabolite profile highlights that over time, as sulfation depletes, this results in increased metabolism via the CYP 450 pathway which eventually depletes available glutathione stores and acute liver injury results.

There are various limitations to this case report including an imprecise time of ingestion, which is required to accurately utilize the acetaminophen treatment nomogram. However, the treating doctors utilized the earliest possible time of ingestion for the nomogram and repeated the acetaminophen concentration to ensure the level was not rising. Both acetaminophen concentrations were well below the treatment line. It is remotely possible she ingested a modified release preparation or had a further ingestion in the ICU. However, the family did not think she had any access to modified-release preparations, and while in the ICU, she was observed by the nursing staff. Secondly, hypotension may have contributed to this patient's clinical picture of acute liver injury and bowel edema. However, the hypotension was rapidly managed, and she did not have persistent or severe shock to cause ischemic hepatitis [38]. Furthermore, the rise in hepatic enzymes observed in hepatic shock in the majority of patients is subtle, whereas this patient developed fulminant hepatic failure and markedly elevated hepatic enzymes consistent with acetaminophen toxicity [38].

## 4. Conclusion

We present a case of fulminant hepatic failure from acetaminophen poisoning despite two initial acetaminophen concentrations well below the treatment line. Delayed gastric emptying from coingested medications resulted in a delayed acetaminophen peak concentration. This case highlights the issue of altered pharmacokinetics that can occur when patients coingest gut slowing agents. In these cases, it is important to consider decontamination even if delayed.

## Data Availability

Data is included as Supplementary Table 1.

## Abbreviations

ALT: Alanine aminotransferase
APAP-Gluc: Acetaminophen-glucuronide
APAP-Sulf: Acetaminophen-sulfate
APAP-Cys: Acetaminophen-cysteine
APAP-Mer: Acetaminophen-mercapturate
AST: Aspartate aminotransferase
AUC: Area under the curve
CYP: Cytochrome P450
INR: International normalised ratio
NAPQI: N-acetyl-p-benzoquinone imine.

## Supplementary Materials

Supplementary Materials Supplementary Table 1: patient's pathology results during admission.
