# Supplementary material for: Delayed Acetaminophen Absorption Resulting in Acute Liver Failure
Source: Case Rep Crit Care. 2022 May 7;2022:3672248. doi: 10.1155/2022/3672248 (PMC9107358; doi:10.1155/2022/3672248)
Supplement: Supplementary Materials — Supplementary Table 1: patient's pathology results during admission. [file 3672248.f1.docx]

**Supplementary Table 1:** Patient’s pathology results during admission

| **Approximate time post-ingestion (hours)** | **4.5** | **8.4** | **16.7** | **41.3** | **47.8** | **51.7** | **60.6** | **67.8** | **72.8** |
| --- | --- | --- | --- | --- | --- | --- | --- | --- | --- |
| **Acetaminophen**  **concentration**  **(µmol/L)** | 256 | 242 | 174* | 560 | 385 | 327 | 250 | 161 |  |
| **Sodium (mmol/L)** | 143 | 146 | 145 | 134 | 138 |  |  | 140 | 142 |
| **Potassium (mmol/L)** | 3.8 | 3.6 | 3.8 | * | 4.4 |  |  | 4.2 | 4.3 |
| **Urea (mmol/L)** | 4.4 | 3.3 | 3.9 | 1.8 | 2 |  |  | 1.9 | 1.8 |
| **Creatinine (µmol/L)** | 44 | 32 | 52 | 37 | 45 |  |  | 116 | 124 |
| **eGFR ml/min/1.73m2** | 119 | 132 | 113 | 126 | 118 |  |  | 50 | 46 |
| **ALP (U/L)** | 61 | 52 | 50 | 67 | 82 |  |  | 107 | 106 |
| **ALT (U/L)** | 20 |  | 15 | 489 | 7009 |  | 5485 | 5120 | 4733 |
| **AST (U/L)** | 25 |  | 20 | * | 16154 |  | 16360 | 18061 | 17042 |
| **GGT (U/L)** | 17 |  | 14 | 28 | 35 |  |  | 39 | 41 |
| **Ammonia** |  |  |  |  |  | 74 |  |  |  |
| **Bili (umol/L)** | < 3 |  | <3 | 58 | 54 |  |  | 77 | 84 |
| **INR** | 1.1 |  |  |  |  | 7.5 | 3.9 | 2.9 | 3.6 |
| **APTT (secs)** | 27 |  |  |  |  |  |  | 45 | 48 |

*Tested retrospectively
